# Insights into memory effect mechanisms of layered double hydroxides with solid-state NMR spectroscopy

**DOI:** 10.1038/s41467-022-33912-7

**Published:** 2022-10-14

**Authors:** Li Jin, Xiaoyuan Zhou, Fang Wang, Xiang Ning, Yujie Wen, Benteng Song, Changju Yang, Di Wu, Xiaokang Ke, Luming Peng

**Affiliations:** 1 Key Laboratory of Mesoscopic Chemistry of MOE, School of Chemistry and Chemical Engineering, Nanjing University, Nanjing, 210023 China; 2 College of Food Science and Engineering/Collaborative Innovation Center for Modern Grain Circulation and Safety/Key Laboratory of Grains and Oils Quality Control and Processing, Nanjing University of Finance and Economics, Nanjing, 210023 China; 3 Jiangsu Key Laboratory of Vehicle Emissions Control, Nanjing University, Nanjing, 210093 China; 4 Frontiers Science Center for Critical Earth Material Cycling (FSC-CEMaC), Nanjing University, Nanjing, Jiangsu 210023 China

**Keywords:** Physical chemistry, Solid-state NMR, Environmental chemistry

## Abstract

Layered double oxides (LDOs) can restore the parent layered double hydroxides (LDHs) structure under hydrous conditions, and this “memory effect” plays a critical role in the applications of LDHs, yet the detailed mechanism is still under debate. Here, we apply a strategy based on ex situ and in situ solid-state NMR spectroscopy to monitor the Mg/Al-LDO structure changes during recovery at the atomic scale. Despite the common belief that aqueous solution is required, we discover that the structure recovery can occur in a virtually solid-state process. Local structural information obtained with NMR spectroscopy shows that the recovery in aqueous solution follows dissolution-recrystallization mechanism, while the solid-state recovery is retro-topotactic, indicating a true “memory effect”. The amount of water is key in determining the interactions of water with oxides, thus the memory effect mechanism. The results also provide a more environmentally friendly and economically feasible LDHs preparation route.

## Abstract (web summary)

The “memory effect” of layered double hydroxides (LDHs) plays a critical role in their applications, yet the details of the mechanism are still under debate. Here authors reveal the nature of the “memory effect” with ex situ and in situ solid-state NMR spectroscopy.

## Introduction

**Fig. 1:**
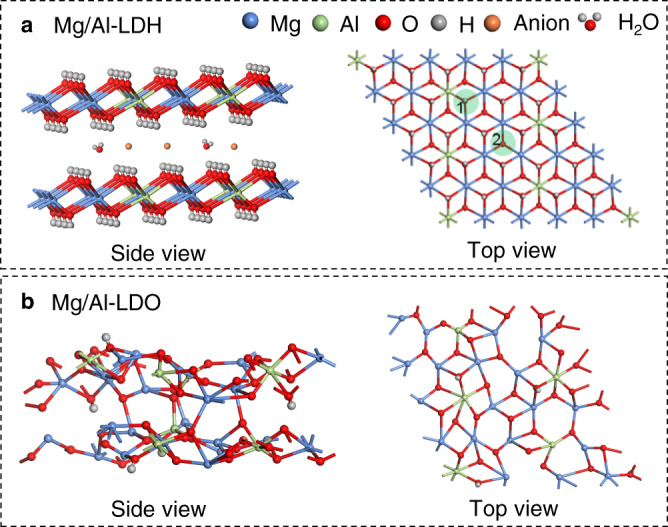
Structure of Mg/Al-LDH and Mg/Al-LDO. Schematic structure of Mg/Al-LDH (**a**) and Mg/Al-LDO (**b**) in the side and top views. The shaded green circles show the local environment of Mg_2_AlOH (1) and Mg_3_OH (2).

Layered double hydroxides (LDHs), a class of bimetallic hydroxide materials, have received a lot of research attention because of their applications in many fields, such as adsorbents, catalysts and energy storage materials^1–5^. One of the most attractive features of LDHs materials is the so-called “memory effect”: layered double oxides (LDOs) formed by calcining LDHs at a moderately high temperature, can restore the original LDHs structure in contact with the aqueous solution containing appropriate anions (Fig. 1)^6–9^. Memory effect has a variety of applications, including pollutant removal^10^, preparations of new LDHs^11^, activation of catalysts^12^, as well as structural investigations of LDHs^13^. It is therefore important to understand the details on the structure change during the LDHs regeneration.

Despite enormous efforts to investigate this issue by applying methods such as X-ray Photoelectron Spectroscopy (XPS), X-ray absorption fine structure spectroscopy (XAFS) and X-ray diffraction (XRD)^14–17^, detailed information on this process is still missing, and the memory effect mechanism remains a topic of intense debate in the literature^18–20^. One of the main reasons behind this controversy is the complicated nature of the structures in the LDHs regeneration process, in which considerable turbostratic disorder and stacking faults in the hydroxide or oxide layers may be present, making structural characterization challenging^21^. Thus, characterization techniques that can provide local environments of LDOs and LDHs are required to fill the missing piece of the puzzle.

Solid state nuclear magnetic resonance (NMR) spectroscopy is a powerful tool with which to provide quantitative, nuclear specific and local structural information of solids at the atomic level^22–26^. Consequently, it should be an ideal approach to give the short-range order of LDOs and LDHs. In principle, ^1^H, ^27^Al, ^17^O, and ^25^Mg NMR spectroscopy can all be used to explore the structure of Mg and Al-containing LDHs; however, ^17^O and ^25^Mg NMR investigations are very challenging and were rarely applied because of their low natural abundances, relatively low gyromagnetic ratios and quadrupolar nature^13,27^. Therefore, ^1^H and ^27^Al solid-state NMR spectroscopy represent more convenient approaches to illustrate the structure of LDHs. Different H species, including Mg_3_OH, Mg_2_AlOH, MgAl_2_OH, and interlayer water in Mg/Al-LDH can be distinguished by using ^1^H NMR spectroscopy^28–30^, and 4- and 6-coordinated Al ions can be easily differentiated with ^27^Al NMR spectroscopy. In this work, we combine ex situ and in situ ^1^H and ^27^Al NMR spectroscopy, and demonstrate this approach can track the detailed structure change during the LDHs regeneration, even in real time. A solid-state recovery scheme is found to be possible, which involve different mechanism compared to recovery in aqueous solutions. The results indicate the importance of the amount of water in controlling the interactions and reactions between oxide and water, which are widely occurring in nature and man-made systems.

## Results

**Fig. 2:**
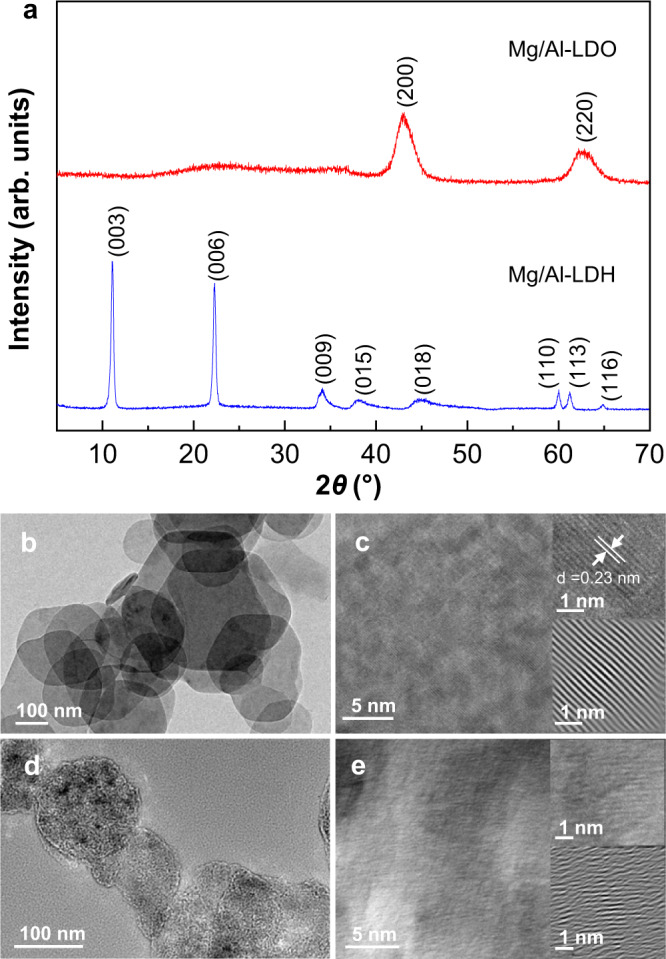
XRD patterns and electron microscopy images of Mg/Al-LDH and Mg/Al-LDO. **a** XRD patterns of Mg/Al-LDH and Mg/Al-LDO. TEM (**b**) and HRTEM (**c**) images of Mg/Al-LDH. TEM (**d**) and HRTEM (**e**) images of Mg/Al-LDO.

Mg- and Al-containing LDH (Mg/Al-LDH) with Al molar ratio of 20 % was prepared by co-precipitation method (Supplementary Table 1)^28,31^, and the corresponding Mg/Al-LDO was obtained by heating Mg/Al-LDH at 450 °C. The material before calcination shows a typical XRD pattern of LDH, and the interlayer distance (*d*_003_ = 0.80 nm) suggests that the sample is a typical NO_3_^−^ intercalated LDH (Fig. 2a)^32^. After calcination, two peaks at 43.2° and 62.6° can be attributed to (200) and (220) reflections of the MgO structure, respectively, while no diffraction peak due to the Al_2_O_3_ phase can be observed, indicating that Al^3+^ ions are well dispersed in the MgO-like structure and Mg/Al-LDO is formed^33^. Fig. 2b–e shows the microstructures and morphologies of the Mg/Al-LDH and Mg/Al-LDO materials. Mg/Al-LDH exhibits a laminar structure, while the well-defined morphology is retained in Mg/Al-LDO.

Due to large ^1^H-^1^H dipolar interactions, deuteration of the samples were performed to decrease the proton density and thus ^1^H homonuclear dipolar coupling, and to improve spectral resolution at medium to low spinning speed in solid-state NMR experiments^28^. High-resolution ^1^H NMR spectra can be obtained on deuterated Mg/Al-LDH samples (Supplementary Fig. 1), and analysis shows that they provide quantitative information on the relative concentration of Mg_3_OH and Mg_2_AlOH species (Supplementary Fig. 2). The ^1^H magic-angle spinning (MAS) NMR spectrum of Mg/Al-LDO shows a weak peak at approx. −0.1 ppm and a shoulder at ~0.9 ppm, both of which can be assigned to residual hydroxyl groups after thermal treatment (Supplementary Fig. 3a)^34^. The ^27^Al MAS NMR spectrum of Mg/Al-LDO exhibits two broad resonances with maxima at ~74 and 10 ppm, corresponding to 4- and 6-coordinated Al ions, which is consistent with the observation in the literature (Supplementary Fig. 3b)^35^.

### Ex situ XRD studies

**Fig. 3:**
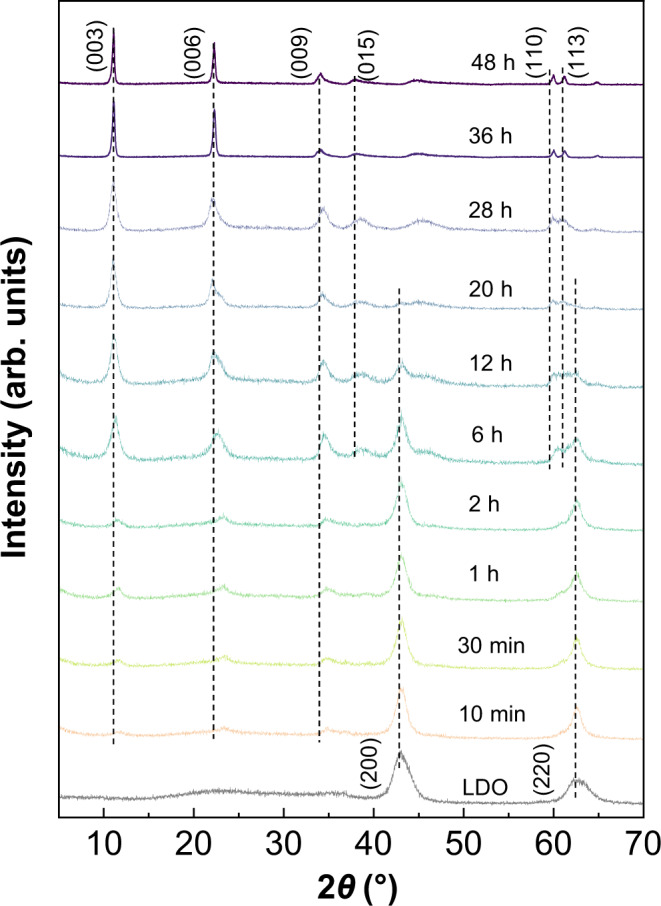
Ex-situ XRD patterns of rehydrated Mg/Al-LDO. XRD patterns of the resulting solids after mixing Mg/Al-LDO with NaNO_3_ solution in D_2_O during a conventional recovery process (mass ratio of Mg/Al-LDO to D_2_O is ~1:100) for a specific rehydration time *t* (10 min to 48 h).

After mixing Mg/Al-LDO with NaNO_3_ solution in D_2_O during a conventional recovery process (mass ratio of Mg/Al-LDO to D_2_O is ~1:100), the ex situ XRD patterns of the resulting solids after a specific rehydration time *t* (10 min to 48 h). are shown in Fig. 3, and the cell parameters refined from this data are given in Supplementary Table 2. The (003) reflection of LDH starts to appear after 10 min, and shifts to lower angles with increasing *t*, suggesting that nitrate anions are gradually intercalated into the interlayer spaces and the distance between lamellae is increased. The intensities of the (003) reflections of the samples with relatively short *t* (10 min to 6 h) are less intense than those of the Mg/Al-LDH and the reflections ((200) and (220)) due to MgO-like phase (LDO) are still present, suggesting that the recovery process is far from being finished and the regenerated LDH phase is not well crystallized at this stage. The peaks owing to LDO become much weaker after *t* = 20 h and disappear *t* = 28 h, suggesting complete conversion of LDO and the reconstruction of the LDH structure is virtually completed, which can be concluded from the similar characteristic diffraction line with the Mg/Al-LDH. The (003) reflection peaks for the sample with a longer *t* (28 to 48 h) become narrower with increasing time, indicating further crystallization of the LDH structure. No peak due to any intermediate species is observed, indicating that the transformation from Mg/Al-LDO to Mg/Al-LDH does not involve another crystalline phase, and such recovery may occur in a single step. TG/DTG profiles and nitrate concentrations show the LDH structure is regenerated after 36 h (see Supplementary Fig. 4, Supplementary Table 3 and related discussion in Supplementary Information), and this recovery time is slightly longer than the time suggested by XRD (28 h). Nonetheless, details in the structure change on molecular level are still missing.

### Ex situ solid-state NMR studies

**Fig. 4:**
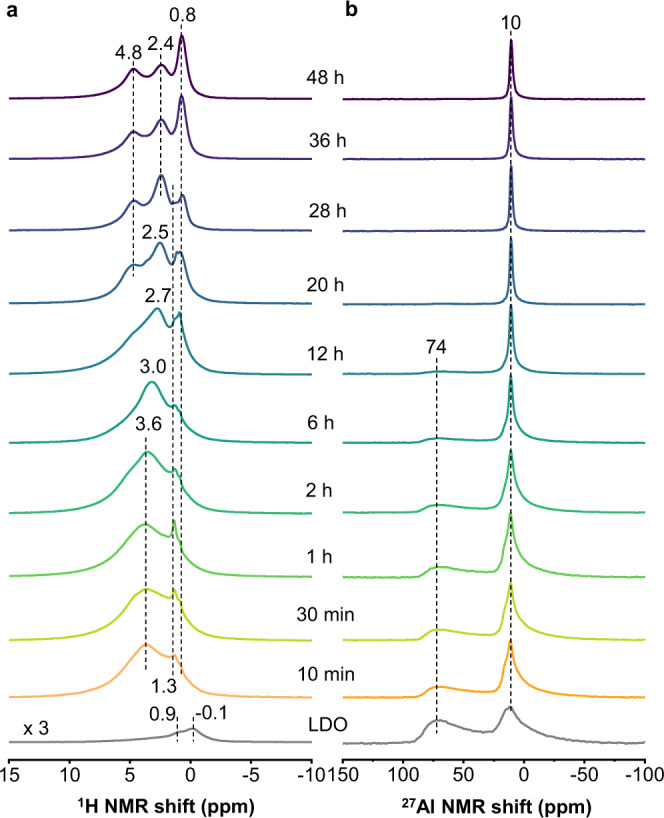
Ex situ NMR spectra of rehydrated Mg/Al-LDO. **a** ^1^H and **b** ^27^Al MAS NMR spectra of rehydrated Mg/Al-LDO for different rehydration time *t*. Mg/Al-LDO was rehydrated with NaNO_3_ in D_2_O solution (mass ratio of LDO to D_2_O is 1:100).

Thus, the corresponding ex situ ^1^H and ^27^Al NMR spectra were also obtained (Fig. 4). The NMR data change dramatically with different rehydration time *t*, implying that NMR spectroscopy is a sensitive method to probe the structure evolution for the memory effect. Even with a short *t* of 10 min, the relative intensity of the peak at 74 ppm in the ex situ ^27^Al NMR spectrum decreases significantly, while the signal at around 10 ppm increases, indicating that a large fraction of 4-coordinated Al ions has been converted to 6-coordinated Al species (Fig. 4b). At the same time, the ex situ ^1^H NMR spectral intensity is significantly stronger than Mg/Al-LDO, indicating that rich hydroxyl groups are generated by water dissociation (Fig. 4a). With increasing *t* (10 min to 2 h), a broad ^1^H resonance with maximum at 3.6 ppm is observed, along with a sharper peak at 1.3 ppm and a shoulder at an even lower frequency (0.8 ppm), indicating multiple ^1^H chemical environments.

The relative intensity of the ex situ ^27^Al NMR peak at 74 ppm further decreases at *t* = 2–20 h while the ^27^Al NMR resonance at 10 ppm becomes narrower, implying that the conversion from 4-coordinated Al to 6-coordinated Al continues during this time. The 4-coordinated Al ions can no longer be observed at 20 h, suggesting that the above conversion process completes. Meanwhile, the ex situ ^1^H NMR resonances show a noticeable change: the signal at 3.6 ppm shifts to 3.0 ppm at *t* = 6 h, and further to 2.7 ppm at *t* = 12 h, which can be attributed to the significant short-range structure changes resulting from the formation of Mg_2_AlOH species in Mg/Al-LDH structure^25^. The resonance at ~0.8 ppm becomes more prominent after 12 h, which can be ascribed to Mg_3_OH species in the Mg/Al-LDH phase. At *t* = 20 h, the ^1^H NMR spectrum is much better resolved, and the major resonances are observed at 0.8–1.3, 2.5 and 4.8 ppm, which can be readily assigned to Mg_3_OH, Mg_2_AlOH and interlayer water in Mg/Al-LDH, respectively^25^.

At a longer rehydration time *t* (28–48 h), ex situ ^27^Al NMR spectra show a single sharp peak at 10 ppm and exhibit no obvious change with time, indicating that all the Al ions are 6-coordinated at this stage. However, the ex situ ^1^H NMR spectra exhibit significant changes. The relative intensities of the peaks at ~0.8 and 2.4 ppm vary at different mixing time. At *t* = 28 h, the peak at 2.4 ppm is the strongest peak, while the resonance at 0.8 ppm becomes the most intense peak in the spectra of the samples with *t* at 36 and 48 h (Supplementary Fig. 5). The increase of the fraction of Mg_3_OH sites in this final stage (*t* = 28–48 h) suggests that more Mg_3_OH species are generated than Mg_2_AlOH, and thus the formation of Mg_3_OH is slower than Mg_2_AlOH in the recovery process. These NMR data are very different from XRD results, which provide long-range order: the XRD reflections owing to LDO disappear after *t* = 28 h and only peaks due to LDH remain (Fig. 3), suggesting complete conversion of LDO to LDH while the NMR results show that the short-range structure is still changing. Furthermore, no sharp component at 1.3 ppm can be observed after 36 h and only one strong peak at 0.8 ppm is present, implying that the peak at 1.3 ppm should correspond to an intermediate species.

**Fig. 5:**
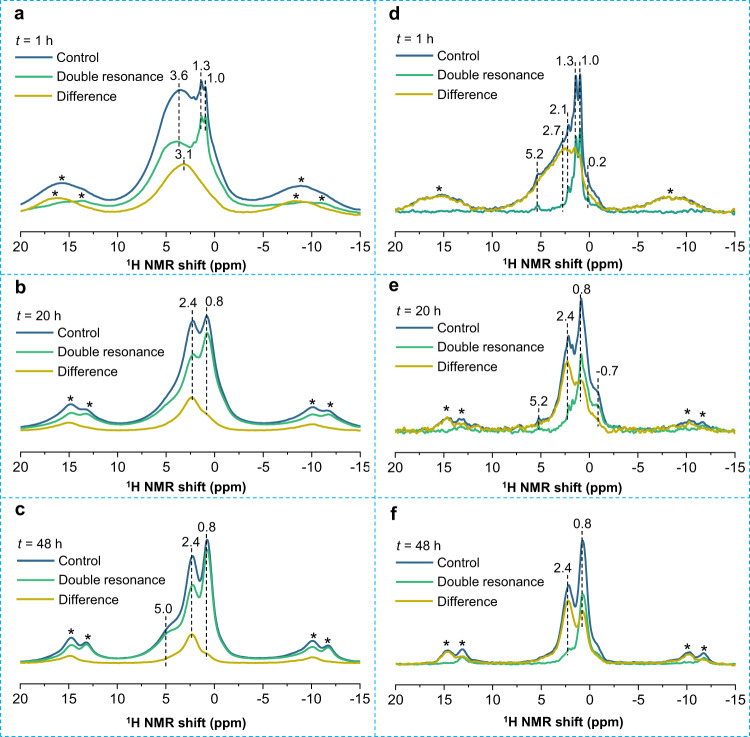
^1^H-^27^Al TRAPDOR NMR data of rehydrated Mg/Al-LDO. ^1^H-^27^Al TRAPDOR NMR spectra of Mg/Al-LDO with a rehydration time *t* = 1 (**a**), 20 (**b**) and 48 h (**c**) and a recoupling time of 0.2 ms. ^1^H-^27^Al TRAPDOR NMR spectra of Mg/Al-LDO with a rehydration time *t* = 1 (**d**), 20 (**e**) and 48 h (**f**) and a recoupling time of 2 ms. Mass ratio of Mg/Al-LDO to D_2_O: ~1:100; spinning speed: 5 kHz.

These ^1^H NMR spectral assignments are further supported by ^1^H-^27^Al TRAnsfer of Population in DOuble Resonance (TRAPDOR) experiments (Fig. 5)^36^. The TRAPDOR control spectrum of Mg/Al-LDO after conventional recovery (*t* = 1 h), shows a broad peak at ~3.6 ppm with relatively sharp components at 1.0 and 1.3 ppm (Fig. 5a). The double resonance spectrum, acquired with continuous irradiation on ^27^Al of 0.2 ms, exhibits significant intensity decrease for the broad peak at 3.1 ppm, indicating that this peak is due to hydroxyl species close to Al ions. On the other hand, the sharp peaks appear in the double resonance spectrum with similar intensity compared to the control spectrum, indicating that these resonances correspond to local environments not very close to Al atoms. According to the chemical shifts, the resonances at 1.0 and 1.3 ppm can be assigned to hydroxyl sites around only Mg atoms. At *t* = 20 h, the control spectrum shows two major resonances at 0.8 and 2.4 ppm (Fig. 5b). The corresponding double resonance spectrum exhibits significant spectral intensity decrease for both the signals at 0.8 and 2.4 ppm, while the latter has a greater effect. Since the LDH structure has been formed at this stage according to diffraction data, the two signals at 0.8 and 2.4 ppm should be ascribed to Mg_3_OH and Mg_2_AlOH sites in hydroxide sheets. The former (0.8 ppm) shows a non-zero TRAPDOR effect due to Al in the 4^th^ coordination shell (i.e., H-O-Mg-O-Al), which increases with longer ^27^Al irradiation time (Fig. 5d–f). Finally, at *t* = 48 h, well-resolved peaks at 2.4 and 0.8 ppm along with a relatively strong shoulder at 5.0 ppm can be observed, corresponding to Mg_2_AlOH and Mg_3_OH in the hydroxide sheets and interlayer water in the well-crystallized LDH structure, respectively (Fig. 5c). According to the optimized LDH structure, the H-Al distances in H-O-Al (Mg_2_AlOH) and H-O-Mg-O-Al (Mg_3_OH) environments are predicted to be 2.5 and 4.2/5.2 Å (Supplementary Fig. 6), which is in agreement with the relatively large and small TRAPDOR fractions for the peaks at 2.4 and 0.8 ppm, respectively.

### In situ solid state NMR studies

**Fig. 6:**
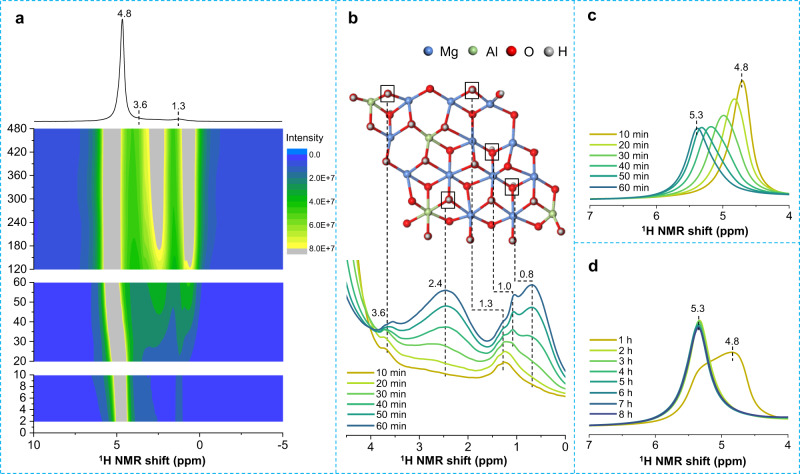
In situ ^1^H NMR spectra of rehydrated Mg/Al-LDO. **a** 2D in situ ^1^H MAS NMR spectra of rehydrated Mg/Al-LDO (mass ratio of LDO to D_2_O is 1:1). The 1D spectrum shown on top corresponds to a rehydration time of 2 min. Each spectrum during rehydration time *t* = 2−10 min, 10 −60 min and 2−8 h takes ~2 min, 10 min and 1 h to collect, respectively, by adjusting the acquisition numbers. **b** Overlay of in situ ^1^H MAS NMR spectra in (**a**) in the range of 0 −4.5 ppm with *t* = 10−60 min along with the corresponding spectral assignments. **c** Overlay of in situ ^1^H MAS NMR spectra in (**a**) in the range of 4−7 ppm with *t* = 10 − 60 min. **d** Overlay of in situ ^1^H MAS NMR spectra in (**a**) in the range of 4−7 ppm with *t* = 1−8 h.

In order to further explore the details of structural changes associated with the “memory effect”, NaNO_3_ solution in D_2_O was added to the NMR rotor packed with Mg/Al-LDO and in situ ^1^H and ^27^Al MAS NMR spectra were acquired to investigate the structure change in real time (Fig. 6 and Supplementary Figs. 7, 8). In this case, the mass ratio of LDO to D_2_O is ~1:1, while this amount of water is enough for the hydroxylation of LDO as well as interlayer water in the LDH structure according to the composition of Mg/Al-LDH (Supplementary Fig. 4 and Supplementary Table 1). Since only a small amount of solution is added, this is a “solid-state recovery” attempt to generate Mg/Al-LDH from Mg/Al-LDO.

In the first 10 min of rehydration, in addition to the major peak at 4.8 ppm due to water, two weak shoulders can be clearly observed at ~3.6 and 1.3 ppm, which are assigned to hydroxyl groups close to both Mg and Al ions, and only Mg ions, respectively, according to the ^1^H NMR shift^25^. Even with a short rehydration time *t* of just 2 min, these two resonances are already present, implying that water molecules dissociate very rapidly to form hydroxyl species at some highly active sites, possibly Lewis sites (Mg^2+^−O^2−^ and Al^3+^−O^2−^ acid-base pairs) at the edge or surface of Mg/Al-LDO^37^. Diffusion of water should also occur in this first 2 min time period, which is probably accelerated by rapid magic angle spinning. In this 10 min, 4-coordinated Al shows little change in the ^27^Al NMR spectra, while 6-coordinated Al signal shows a slight increase, indicating that some Al species in Mg/Al-LDO, which are not detected due to large quadrupolar interactions, are converted to 6-coordinated Al ions during this time (Supplementary Fig. 8).

With a longer rehydration time *t* of 10 to 60 min, broader peaks with peak maxima at ~2.4 and 0.8 ppm emerge and increase significantly with time, implying that the occurrence of a continuous water dissociation process. These characteristic resonances can be readily assigned to Mg_2_AlOH and Mg_3_OH species in the hydroxide sheets of the newly formed Mg/Al-LDH structure, respectively^25^. XRD pattern of the sample right after collecting the in situ ^1^H NMR spectrum at *t* = 60 min confirms the presence of LDH phase in addition to the LDO phase at this stage (Supplementary Fig. 9). In contrast, the intensities of the relatively sharp components at 3.6 and 1.3 ppm do not change much during this period of time. The results imply that these species are intermediate and are further converted to Mg_2_AlOH and Mg_3_OH in the Mg/Al-LDH structure, while new hydroxyl sites on the edge/surface of Mg/Al-LDO are formed at the same time, resulting in nearly unchanged intensity for such signals. Another relative narrow peak at 1.0 ppm, presumably due to Mg_3_OH species in a slightly different environment, can also be observed with *t* > 40 min. Furthermore, the peak due to water gradually shifts to a higher frequency of 5.3 ppm with time. Because water molecules in the interlayer regions are known to resonate at 4.8 ppm^25^, such shift to a higher frequency indicates that more water molecules resonate at a higher frequency, presumably due to hydrogen bonding to the hydroxyl species^38–40^. For in situ ^27^Al MAS NMR spectra, the intensity of the peak at. 74 ppm decreases, while a significant intensity growth and linewidth narrowing can be observed for the peak at ca. 10 ppm, indicating the formation and further crystallization of the Mg/Al-LDH phase.

When the rehydration time is extended from 60 to 480 min, continuous and simultaneous increase in the intensities of the broad peaks centered at ~2.4 and 0.8 ppm can be observed before 360 min, indicating the further generation of the Mg_2_AlOH and Mg_3_OH species in the Mg/Al-LDH structure. However, there is very little change in spectral intensity after 360 min for the peaks arising from Mg_2_AlOH and Mg_3_OH (Supplementary Figs. 10, 11), indicating that the dissociation of water has completed and the vast majority of the LDO structure is converted to LDH during the first 6 h. After *t* = 6 h, almost no signal arising from 4-coordinated Al ions can be observed in in situ ^27^Al NMR spectra (Supplementary Fig. 8). The disappearance of the LDO phase is confirmed by the XRD data on the sample right after obtaining its in situ NMR spectrum at *t* = 6 h (Supplementary Fig. 9). To the best of our knowledge, it is the first example that LDO can restore the parent LDH structure with a very small amount of solution in a virtually solid-state process, while a large amount of water was always considered necessary for such structure conversion form LDO to LDH.

### The nature of memory effect

**Fig. 7:**
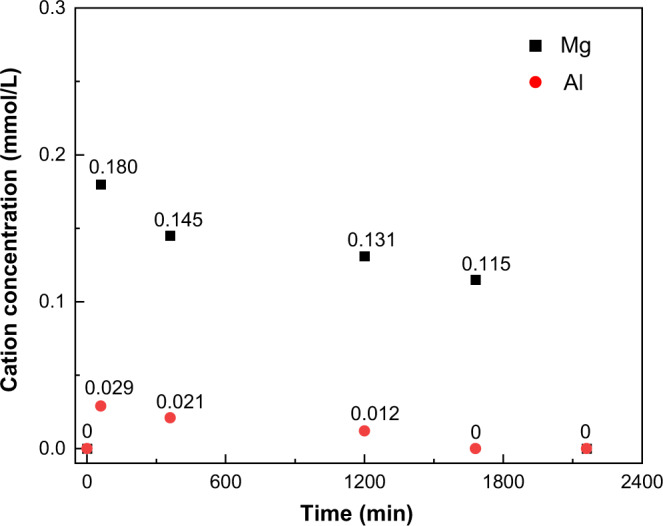
The cation concentration changes in the supernatant solution. The cation concentration in the supernatant solution after centrifugation of the sample in a conventional recovery with rehydration time *t* of 0 min, 60 min, 360 min (6 h), 1200 min (20 h), 1680 min (28 h) and 2160 min (36 h).

By applying solid-state NMR spectroscopy, the details in the structural evolution from LDO to LDH are revealed, even in real time with in situ spectroscopy. Since both the variations of local structure (i.e., Mg_3_OH *vs* Mg_2_AlOH) and recovery time in conventional recovery in acqueous solution and solid-state recovery are significantly different, distinct mechanisms should be involved in the two recovery processes. It is worth noting that our solid-state recovery was performed in an NMR rotor spinning at a very high frequency (20 kHz), therefore, LDO rehydration (mass ratio of Mg/Al-LDO to D_2_O is ~1:1) was also performed at a zero MAS rate (Supplementary Fig. 12). Similar XRD patterns corresponding to LDH structure are observed for the two samples with *t* = 6 h, suggesting that MAS does not account for the faster recovery in the solid-state. Because the relative concentrations of Mg_3_OH and Mg_2_AlOH significantly change during conventional recovery (Fig. 4), the concentrations of Mg and Al ions in the supernatant solution after centrifugation of the sample were measured. Relatively high concentrations of Mg and Al ions are found in the solution in the early and middle stage of rehydration in conventional recovery, while the concentrations of Al and Mg ions only decrease to 0 in the final stage with *t* = 28 h or longer (Fig. 7 and Supplementary Table 4). Thus, the formation of LDH in conventional recovery should be ascribed to the partial dissolution of LDO (in a large amount of water) followed by recrystallization of LDH from the solution. This two-step process is expected to be more time-consuming than a direct structure conversion in solid-state recovery. It is also clear that Mg ions in the LDO dissolve more easily compared to Al ions, and the ratios of Mg to Al in the solution (>6) is always higher than the original ratio in LDO (Mg/Al = 4) before the conversion is finished, resulting in partially recovered Al-rich solid material, therefore, a stronger peak at 2.4 ppm for Mg_2_AlOH than the peak at 0.8 ppm for Mg_3_OH (e.g., *t* = 28 h, Supplementary Fig. 5).

**Fig. 8:**
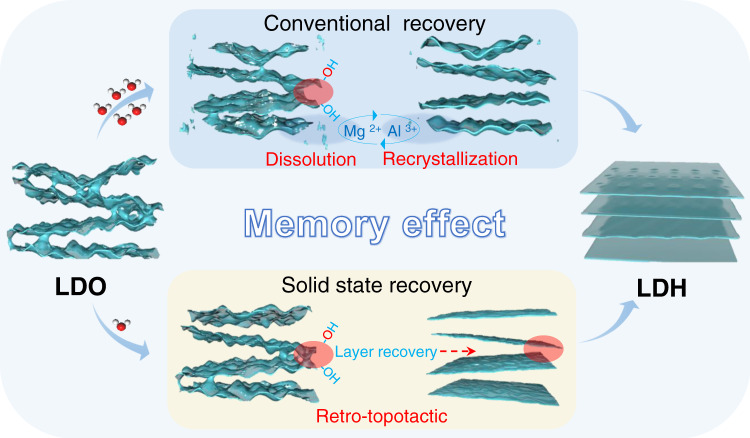
Schematic representation of memory effect mechanisms. The above route from LDO to LDH represents conventional recovery with a large amount of water, in which dissolution occurs first, followed by recrystallization of LDH structure. In the bottom route, LDO structure is regenerated more rapidly in a retro-topotactic process with the presence of a small quantity of water.

The detailed mechanisms of the memory effect of LDH at the atomic scale can be summarized in Fig. 8. When the mass ratio of LDO to D_2_O is ~1:100, the interactions of water with oxides are dominated by the dissolution process and both Mg and Al ions are dissolved, while the Mg^2+^ concentration is higher in solution. Therefore, in the LDH recrystallization process, the formation of Mg_3_OH sites is slower than the Mg_2_AlOH sites, and the whole structure recovery process takes long time (~36 h, Fig. 4 and Supplementary Fig. 13) to finish with such dissolution-recrystallization mechanism. In a solid-state recovery process with the mass ratio of LDO to D_2_O of ~1:1, water molecules can rapidly dissociate on the high active sites to form intermediate hydroxyl species. With enough water molecules for the presence in the interlayer region in LDH, the continuous opening of the interlayer space can be realized, followed by the formation of Mg_2_AlOH and Mg_3_OH environments in the LDH structure. This structure restoration from LDO to LDH should be associated with a retro-topotactic process, leading to a much shorter recovery time of ~6 h. Therefore, we believe that the solid-state recovery represents a true “memory effect” in which both long-range order and short-range order are recovered for LDH, while the conventional recovery process may not regenerate the original local structure (e.g., the distribution and ordering of cations due to the much less controlled dissolution and recrystallization in aqueous solution).

**Fig. 9:**
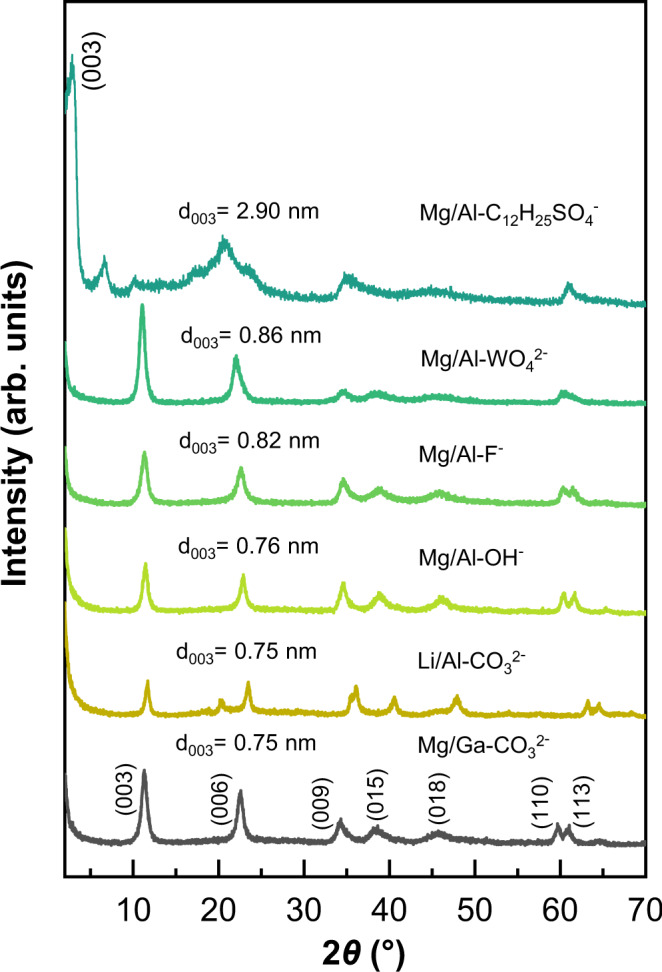
XRD patterns of regenerated LDHs. XRD patterns of LDHs with different anions or cations prepared with a solid-state recovery process from corresponding LDOs.

Compared to the conventional recovery, the solid-state recovery is much faster and only requires a very small amount of water, thus produces much less waste, it may provide a more environmentally friendly and economically feasible way for preparing LDH materials from LDOs^41,42^. Therefore, the preparation of a variety of LDHs with different cations in the hydroxide sheets or interlayer anions have been attempted with this approach (Fig. 9 and Supplementary Fig. 14). The XRD patterns show that the conversion from LDOs to LDHs can be completed successfully in solid state. Therefore, this approach provides a green and robust method for the regeneration of LDH materials.

## Discussion

In conclusion, the structure evolution of the memory effect from Mg/Al-LDO to Mg/Al-LDH can be successfully probed by applying ex situ and in situ solid-state NMR spectroscopy. Differences in the structure regeneration time and the formation order of different hydroxyls species (Mg_3_OH and Mg_2_AlOH) are found to be associated with the amount of water present in the structure reformation process. The conventional structural regeneration route in aqueous solution is associated with a dissolution-recrystallization mechanism, while the solid-state recovery is a direct retro-topotactic process, leading to a true “memory effect” and a much faster structure recovery of the latter. These results help to reconsider the role of water and show that the amount of water may be very important in controlling the interactions between water and oxides, thus the dominating memory effect mechanism for LDH. The characterization approach demonstrated in this study can be readily extended to investigate the details of the interactions of water with other oxides, and many other processes in which the different amounts of water may result in different mechanisms. Extension of this solid-state recovery of LDH from LDO can also provide a green preparation route for a variety of LDHs.

## Methods

### Preparation of the Mg/Al-LDH and Mg/Al-LDO

The Mg/Al-LDH with a Mg/Al molar ratio of 4.0 was prepared by co-precipitation method. Briefly, solution A was obtained by dissolving 0.016 mol Mg(NO_3_)_2_·6H_2_O and 0.004 mol Al(NO_3_)_3_·9H_2_O in 40 mL deionized water. Solution B was prepared by dissolving 0.04 mol NaOH in 40 mL deionized water. The two solutions were then added drop by drop to a 250 mL flask under vigorous stirring at room temperature. At the same time, 0.5 M NaOH or 0.5 M HNO_3_ solution was added dropwise to the mixture to maintain a pH of 10, which was monitored with a pH electrode (Mettler Toledo S40K). The mixture was then transferred to a 100 mL Teflon hydrothermal autoclave and heated at 180 °C for 24 h. After that, the product was centrifuged and washed with deionized water until the solution was neutral, and finally dried at 60 °C to obtain Mg/Al-LDH. Mg/Al-LDH was further calcined at 450 °C for 4 h and the product is denoted as Mg/Al-LDO.

### Deuteration of Mg/Al-LDH

The as-synthesized Mg/Al-LDH was suspended in D_2_O for deuteration. The mixture was stirred magnetically at 300 K for 48 h, before it was centrifuged and finally vacuum-dried at 333 K.

### Basic characterization

The powder XRD data were obtained on a Shimadzu XRD-6000 diffractometer using Cu Kα radiation (λ = 1.5418 Å) operating at 40 kV and 40 mA, with a scan step of 5° in the 2θ range from 2° (or 5°) to 70°. Transmission electron microscopy (TEM) images were obtained using a JEM-2010 electron microscope (JEOL, Tokyo, Japan) at 200 kV, where the samples were dispersed by ultrasonic oscillation. The Mg and Al molar percentages were determined by inductively coupled plasma (ICP) emission spectroscopy (Perkin-Elmer ICP OPTIMA-5300DV). The N (nitrate) contents were determined by elemental analysis (Elementar Vario MICRO). The thermogravimetric profile of each sample was collected on a thermal analysis system (NETZSCH STA 449 C) in a N_2_ atmosphere at a ramping rate of 10 °C/min from 30 to 700 °C.

### Solid-state NMR experiments

Solid-state NMR experiments at 9.4 T were performed using a Bruker Avance III spectrometer and a 3.2 mm double resonance probe. ^1^H MAS NMR spectra was collected with a rotor-synchronized spin echo sequence (π/2−τ − π − τ − acq, τ = one rotor period) at a MAS rate of 20 kHz with a recycle delay of 2 s. ^27^Al MAS NMR spectra were obtained with a single pulse sequence with ^1^H decoupling during acquisition at a MAS rate of 20 kHz with a recycle delay of 0.2 s. ^1^H and ^27^Al shifts were referenced to external standards of H_2_O and 0.1 M Al(NO_3_)_3_ aqueous solution at 4.8 and 0.0 ppm, respectively. The ^1^H/^27^Al TRAPDOR (TRAnsfer of Population in DOuble Resonance) experiments were carried out at a spinning speed of 5 kHz and a recoupling time of 0.2 and 2 ms with an rf field of 60 kHz for ^27^Al irradiation. All NMR spectra were corrected for this background resonance by subtracting the empty rotor spectrum.

### Ex situ XRD/NMR experiments

Ex situ XRD/NMR experiments were performed on Mg/Al-LDO samples with different rehydration time *t* at about 300 K (room temperature). Briefly, 100 mg Mg/Al-LDO was added to 10 mL NaNO_3_ solution in D_2_O (mass ratio of Mg/Al-LDO to D_2_O: ~1/100), and after a specific time *t* the solid was recovered by centrifugation before it was washed with deionized water or D_2_O.

### In situ solid-state NMR experiments

For in situ solid-state NMR experiments, 12 mg Mg/Al-LDO was packed in a zirconia rotor before 12 μL NaNO_3_ solution in D_2_O was added to the rotor (solid/liquid: ~1/1), which is followed by in situ NMR investigations. The rotor was spun at 20 kHz for in situ NMR data acquisition (temperature is measured as ~303 K at a MAS rate of 20 kHz). Considering the time (~2 min) for putting the rotor in the probe and tuning the instrument ready for data collection, the first ^1^H NMR spectrum was obtained with a rehydration time t of 2– 4 min. After that, ^1^H NMR spectra were acquired every 2, 10, and 60 min in the first 10 min, 10–60 min, and after 60 min, respectively.

## Supplementary information

Supplementary Information

## Data Availability

All data supporting the findings presented here are included in the manuscript, its supporting information, or from the authors upon request.
