# Supplementary material for: Insights into memory effect mechanisms of layered double hydroxides with solid-state NMR spectroscopy
Source: Nat Commun. 2022 Oct 14;13:6093. doi: 10.1038/s41467-022-33912-7 (PMC9568524; doi:10.1038/s41467-022-33912-7)
Supplement: Supplementary file 1 — Supplementary Information [file 41467_2022_33912_MOESM1_ESM.pdf]

Supplementary Information for

Insights into memory effect mechanisms of layered double hydroxides with solid-state NMR spectroscopy

Jin et al.

Table of contents

Supplementary Methods 3

Supplementary Discussion 5

Supplementary Figures and Tables 7

Supplementary References.......... 23

Supplementary Methods

Chemicals

NaOH (98 %), HNO_3 (65.0-67.0 %), $Al(NO_3)_3·9H_2O$ (98 %), $Mg(NO_3)_2·6H_2O$ (99 %), $LiNO_3·3H_2O$ (99 %), $Ga(NO_3)_3·9H_2O$ (99 %), $NaNO_3$ (99 %), Na_2CO_3 (99 %), NaF (99 %), $Na_2WO_4·2H_2O$ (99 %), $CH_3(CH_2)_{11}OSO_3Na$ (99 %), $CO(NH_2)_2$ (99 %) and D_2O (99.9 % ^2H) were purchased from Sigma-Aldrich and used without further purification. All the solutions were prepared with deionized water.

Preparation of Mg/Ga-LDH, Li/Al-LDH, Mg/Ga-LDO and Li/Al-LDO

Briefly, 0.016 mol $Mg(NO_3)_2·6H_2O$ (or $LiNO_3·3H_2O$), 0.004 mol $Ga(NO_3)_3·9H_2O$ (or $Al(NO_3)_3·9H_2O$) and 0.024 mol $CO(NH_2)_2$ were mixed and transferred to a 100 mL Teflon hydrothermal autoclave and heated at 100 °C for 24 h; after that, the product was centrifuged and washed with deionized water until the solution was neutral, and finally dried at 60 °C to obtain Mg/Ga-LDH (or Li/Al-LDH). Mg/Ga-LDH (or Li/Al-LDH) was further calcined at 450 °C (or 300 °C for Li/Al-LDH) for 4 h and the product was denoted as Mg/Ga-LDO (or Li/Al-LDO).

Preparation of LDHs with solid-state recovery

Briefly, 30 mg Mg/Al-LDO (or Mg/Ga-LDO, Li/Al-LDO) was packed in the 4 mm zirconia rotor before 30 μL water or solution containing stoichiometric amount of Na_2CO_3 (or NaF, $Na_2WO_4·2H_2O$, $CH_3(CH_2)_{11}OSO_3Na$) was added to the rotor. The rotor was kept at 300 K for a specific time of 24 h to obtain the corresponding LDH material.

Computational methods

The structure model of Mg/Al-LDH (Mg/Al = 4) was built within a refined cubic cell (a = b = 15.4 Å and c = 8.0 Å) ,which was obtained from the XRD data. Interlayer water molecules are not considered in this calculation. The geometry-optimized model was performed using the Vienna Ab Initio Simulation Package (VASP).[1] The electron exchange and correlation were modeled within the generalized gradient approximation (GGA) method and the Perdew-Burke-Ernzerhof (PBE) function.[2] The geometry-optimized calculation was performed at the cutoff energy of 500 eV and a 2 × 2 × 3 mesh of K-points. The convergences for the electronic energy and the force are 1×10^{-5} eV and 0.03 eV /Å, respectively.

Supplementary Discussion

^{1}H and ^{27}Al MAS NMR spectra of Mg/Al-LDH

^{1}H MAS NMR spectrum of Mg/Al-LDH shows a broad resonance at approx. 1.6 ppm and a sharper peak at 4.8 ppm, while much narrower peaks can be observed for deuterated Mg/Al-LDH (Supplementary Fig. 1a). The signals at 4.8, 2.4 and 0.8 ppm can be readily assigned to interlayer water, Mg_2AlOH and Mg_3OH, respectively.[3] According to the fitted spectrum of deuterated sample, the molar percentages of Mg_2AlOH (0.592) and Mg_3OH (0.408) can be obtained, which agree well with the predictions based on nonrandom Mg/Al distribution and the avoidance of Al-O-Al linkages, as well as previous investigations.[3] The ^{27}Al MAS NMR spectrum of Mg/Al-LDH exhibits one single sharp resonance at 10 ppm due to 6-coordinated Al ions in the hydroxide sheets, while no difference was observed after deuteration (Supplementary Fig. 1b), indicating that deuteration does not change the structure of LDH.

TG/DTG analyses of Mg/Al-LDH

Supplementary Fig. 4 shows the TG-DTG curves of Mg/Al-LDH and the resulting solids after mixing Mg/Al-LDO with $NaNO_3$ solution in D_2O for a specific rehydration time *t* (6, 12, 20, 28 and 36 h). Decomposition of Mg/Al-LDH reveals four stages in a temperature range of approx. 80 to 550 °C. The first stage at around 156 °C is attributed to the removal of the surface absorbed and interlayer water.[4] The following two stages at approx. 351 and 410 °C correspond to the dehydroxylation of the LDH sheets, presumably from the removal of Mg_2AlOH and Mg_3OH species, respectively.[4] The last stage centered at 475 °C is due to the decomposition of NO_3^- in the interlayer space.[5] However, different features can be observed for the TG/DTG profiles of samples recovered from LDO with relatively short rehydration times

(t = 6, 12, 20 and 28 h). For example, only one peak can be observed at 300 to 450 °C for the rehydrated samples with t = 6 ~ 28 h, indicating the structure is not fully regenerated. With a long enough rehydration time (t = 36 h), two distinct peaks can be observed again in the DTG curve, which is very similar to the original Mg/Al-LDH, confirming that the recovery process is completed at 36 h.

Elemental analysis

The Mg and Al molar percentages of Mg/Al-LDH were determined as 80.2 and 19.8%, respectively, by inductively coupled plasma (ICP) emission spectroscopy (Supplementary Table 1). The nitrate contents of Mg/Al-LDH and the resulting solids after mixing Mg/Al-LDO with $NaNO_3$ solution in D_2O for a specific rehydration time t (6, 12, 20, 28 and 36 h) also reflect the degree of structure recovery (Supplementary Table 3). The nitrate concentration for the rehydrated sample with a short t of 6 h is much smaller than the original Mg/Al-LDH, while the nitrate fraction increases with increasing rehydration time t. The nitrate concentration is practically the same in the rehydrated sample at t = 36 h with the original Mg/Al-LDH, suggesting a full structural recovery.

Supplementary Figures

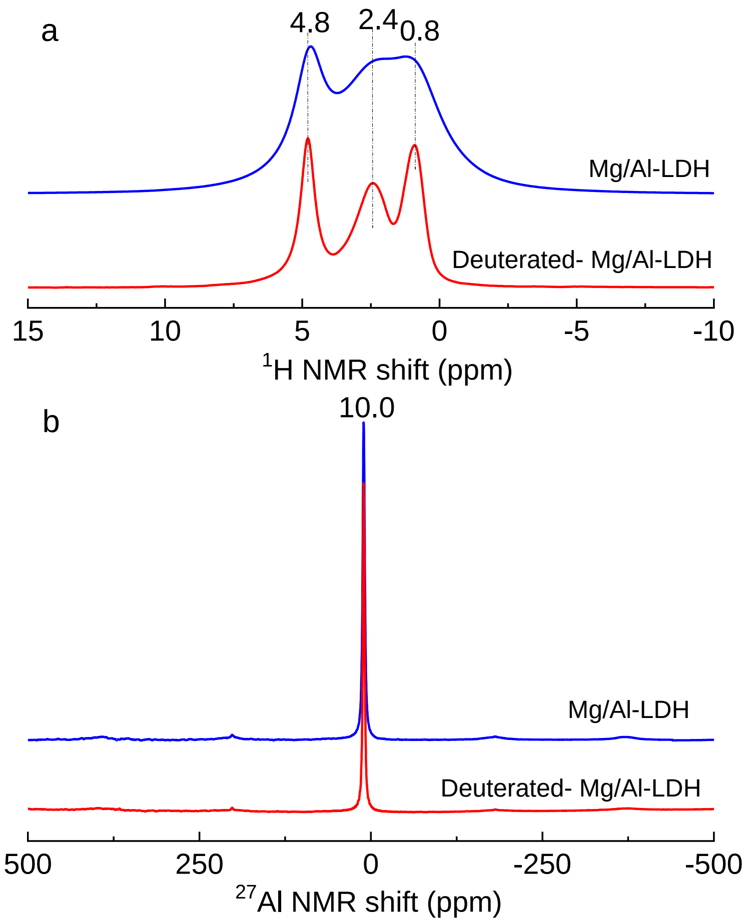

Supplementary Figure 1. ^{1}H NMR spectra (a) and ^{27}Al NMR spectra (b) of Mg/Al-LDH and deuterated Mg/Al-LDH.

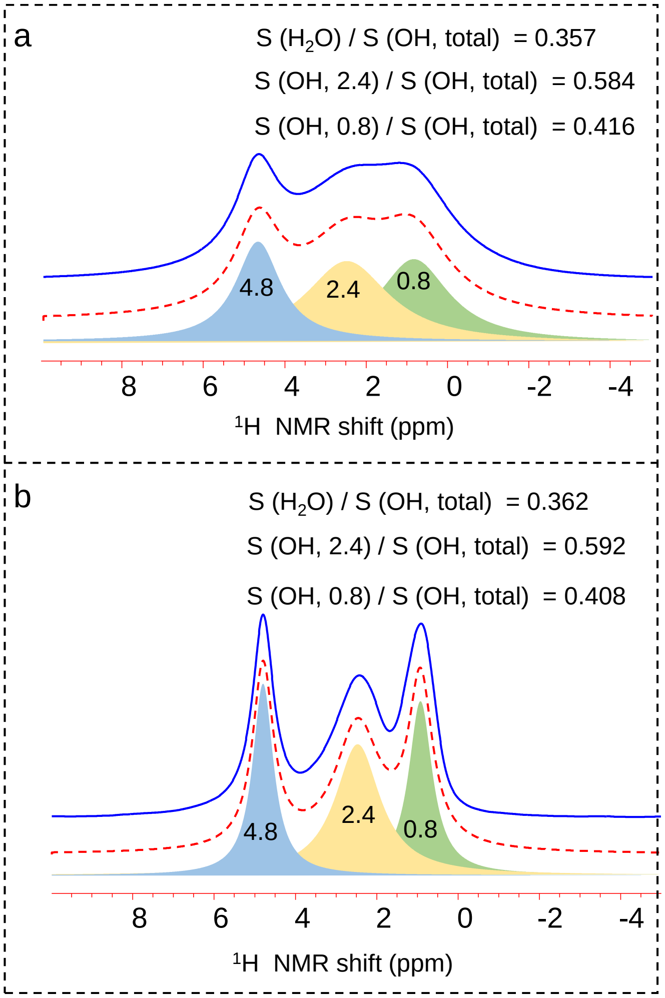

Supplementary Figure 2. ^{1}H NMR spectrum and corresponding spectral deconvolution of (a) Mg/Al-LDH and (b) deuterated Mg/Al-LDH. It is clear that the spectral resolution is much improved by deuteration. The fractions of the intensities for the three peaks due to interlayer water, Mg_2AlOH and Mg_3OH are very similar in both Mg/Al-LDH and deuterated Mg/Al-LDH, indicating that quantitative ^{1}H NMR analysis can still be made with deuteration.

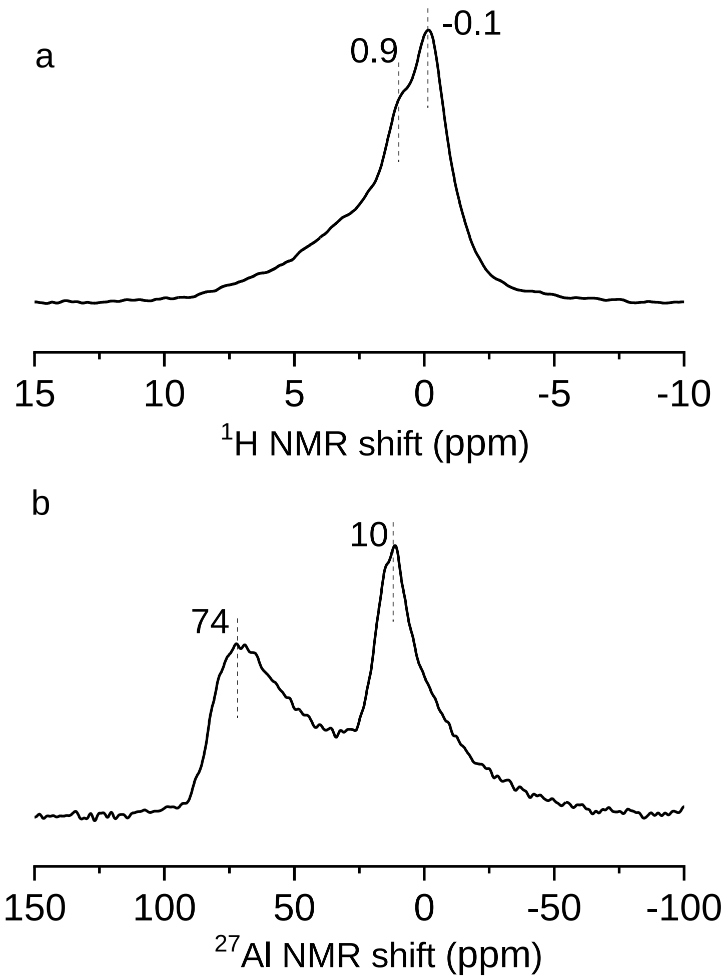

Supplementary Figure 3. ^{1}H NMR spectra (a) and ^{27}Al NMR spectra (b) of Mg/Al-LDO.

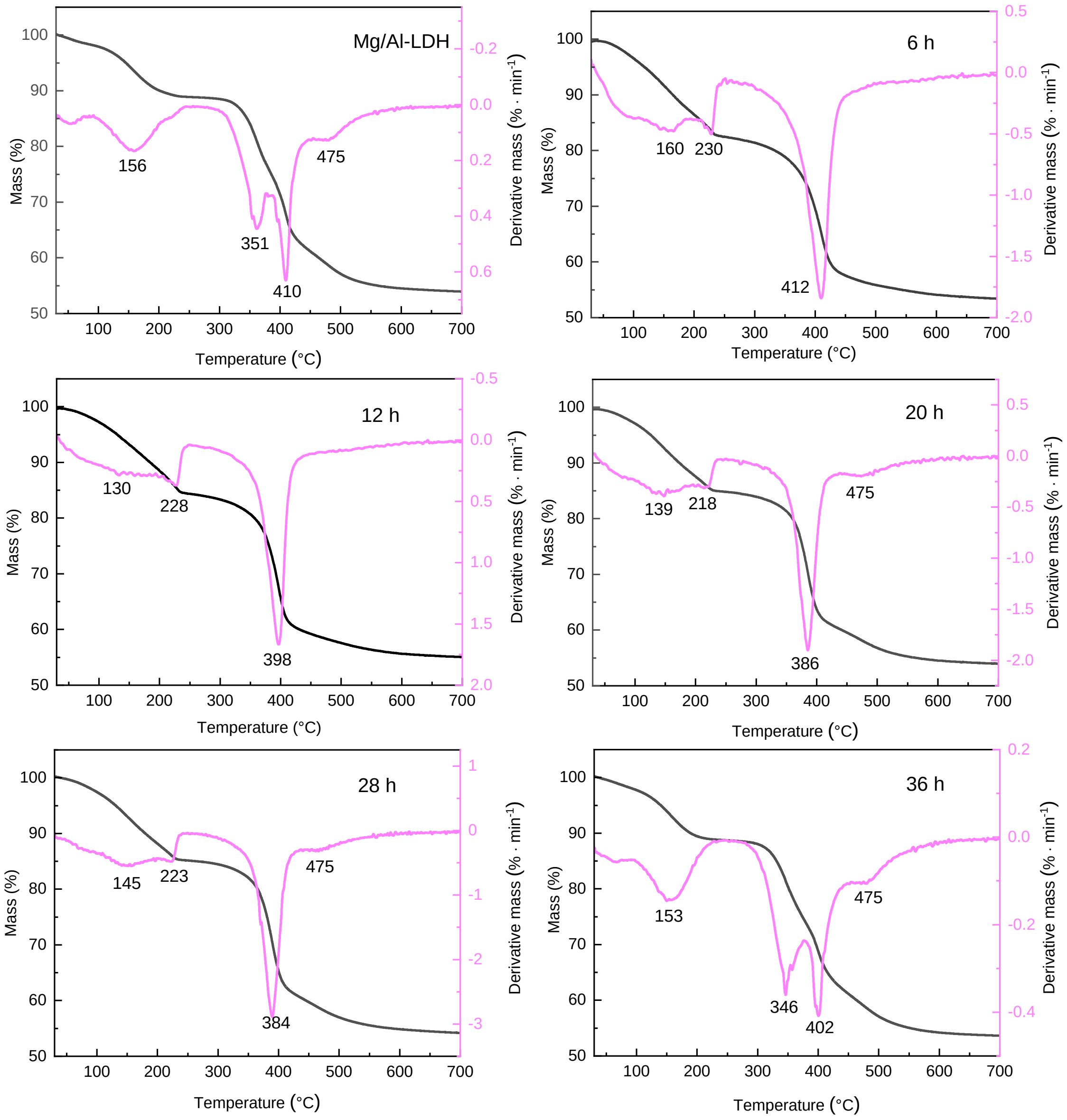

Supplementary Figure 4. TG/DTG curves of Mg/Al-LDH and Mg/Al-LDO rehydrated for different rehydration time *t* (6, 12, 20, 28 and 36 h). Mg/Al-LDO was rehydrated with $NaNO_3$ in D_2O solution (mass ratio of LDO to D_2O is 1:100).

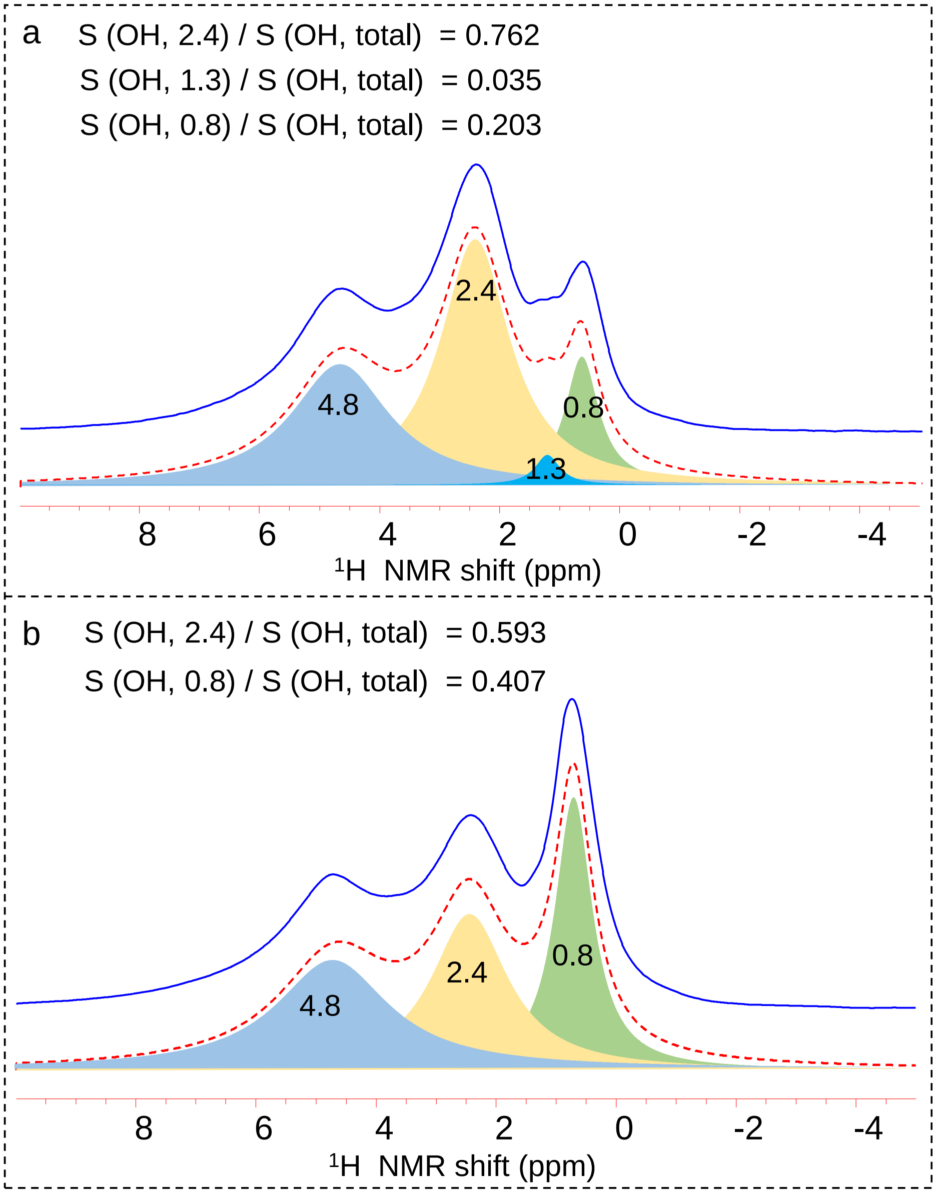

Supplementary Figure 5. ^{1}H NMR spectrum and corresponding spectral deconvolution of (a) Mg/Al-LDO with rehydration time t = 28 h and (b) t = 48 h. Mg/Al-LDO was rehydrated with $NaNO_3$ in D_2O solution (mass ratio of LDO to D_2O is 1:100).

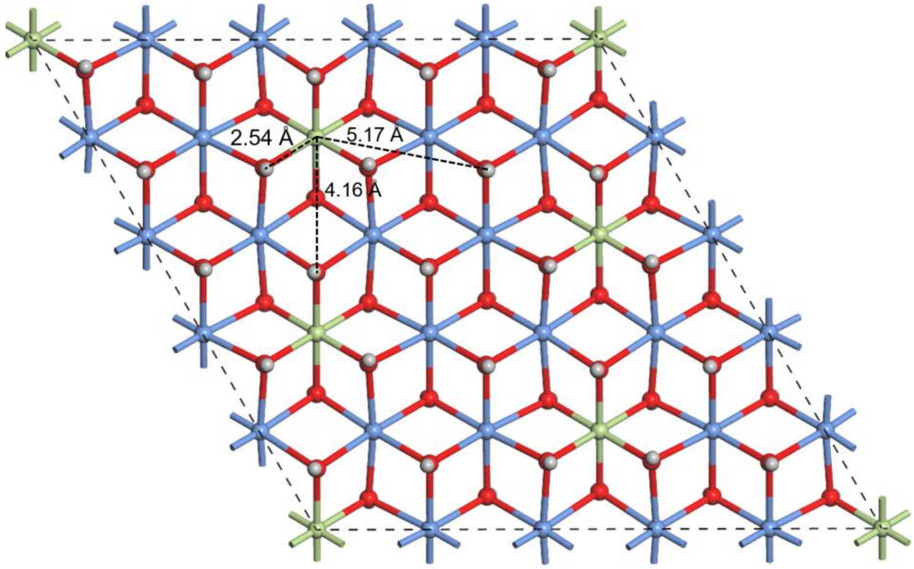

Supplementary Figure 6. The optimized LDH structure (Mg/Al = 4) showing H-Al distances of H-O-Al (Mg_2AlOH) and H-O-Mg-O-Al (Mg_3OH).

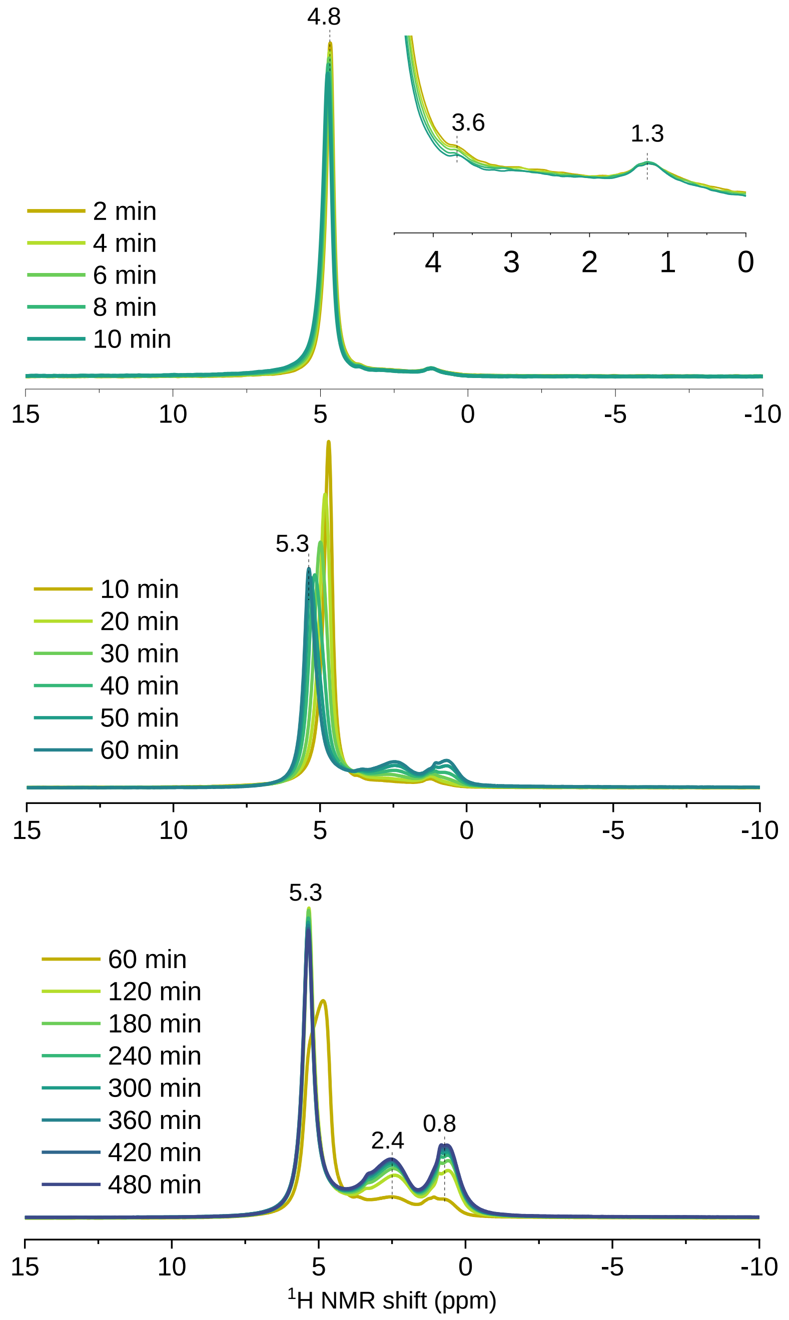

Supplementary Figure 7. In situ ^{1}H MAS NMR spectra of Mg/Al-LDO rehydrated with $NaNO_3$ solution in D_2O (mass ratio of LDO to D_2O is 1:1), the background signals from the probe and the empty rotor are removed. Each spectrum during t = 2 - 10 min, 10 - 60 min and 60 - 480 min takes approx. 2 min, 10 min and 1 h to collect, respectively, by adjusting the scan numbers.

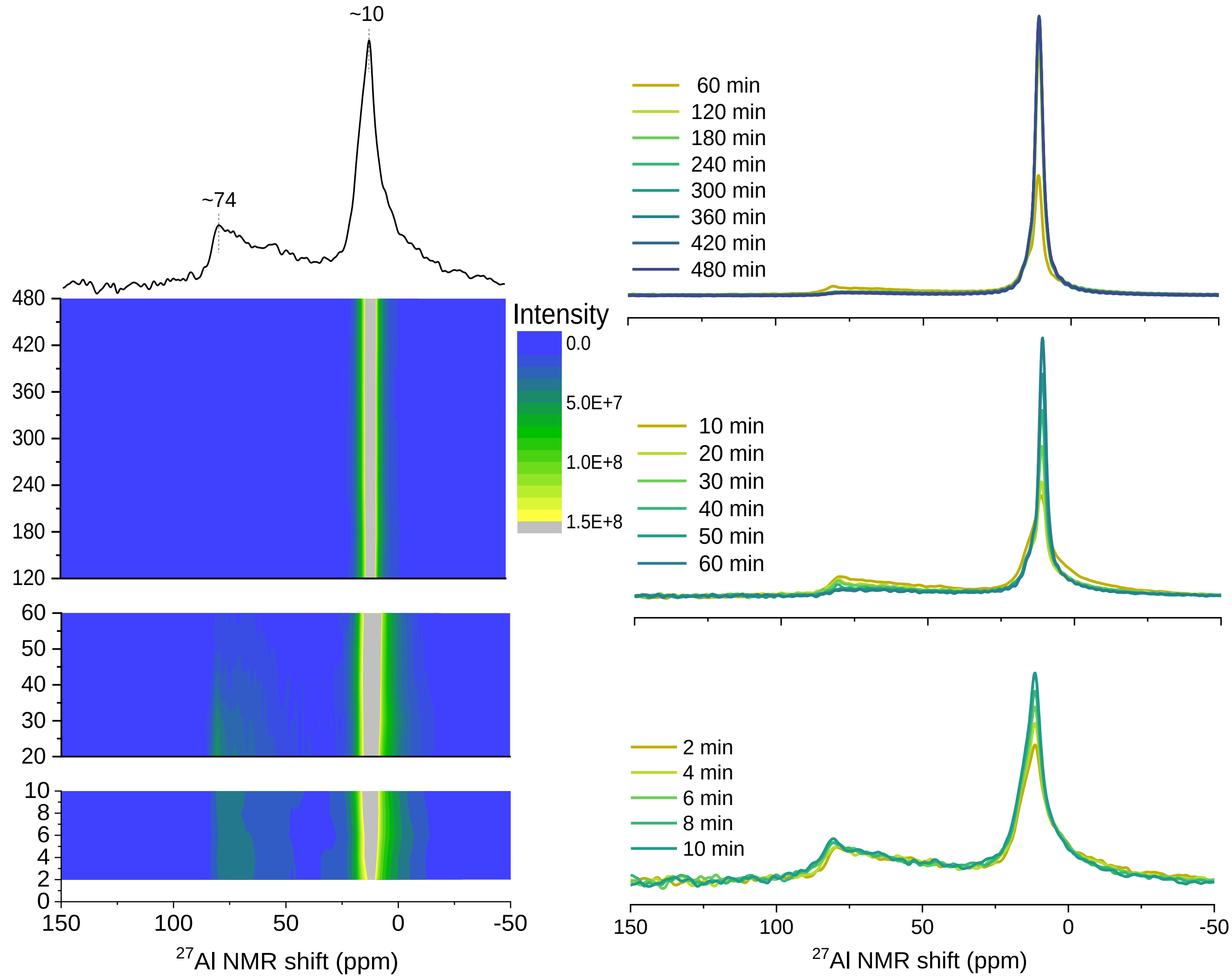

Supplementary Figure 8. In situ ^{27}Al MAS NMR spectra of Mg/Al-LDO rehydrated with $NaNO_3$ solution in D_2O (mass ratio of LDO to D_2O is 1:1), the 1D spectrum shown on top corresponds to the data with *t* of 2 min. The background signals from the probe and the empty rotor are removed. Each spectrum during *t* = 2 - 10 min, 10 - 60 min and 60 - 480 min takes approx. 2 min, 10 min and 1 h to collect, respectively, by adjusting the scan numbers.

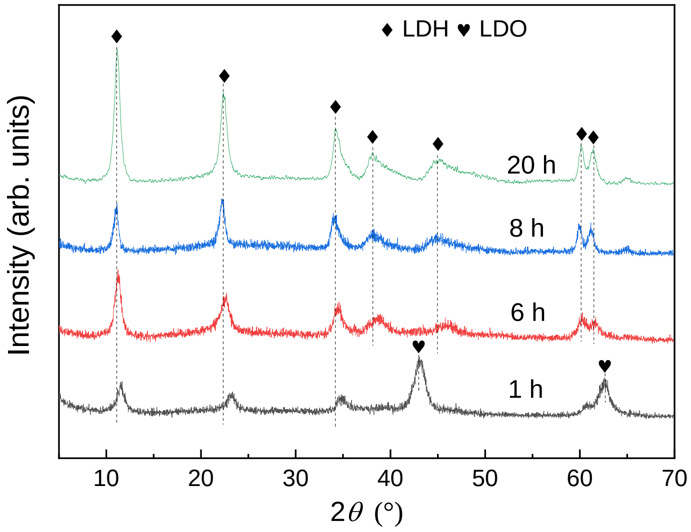

Supplementary Figure 9. XRD patterns of the samples right after collecting the in situ ^{1}H NMR spectrum at t = 1, 6, 8 and 20 h (mass ratio of LDO to D_2O is approx. 1:1).

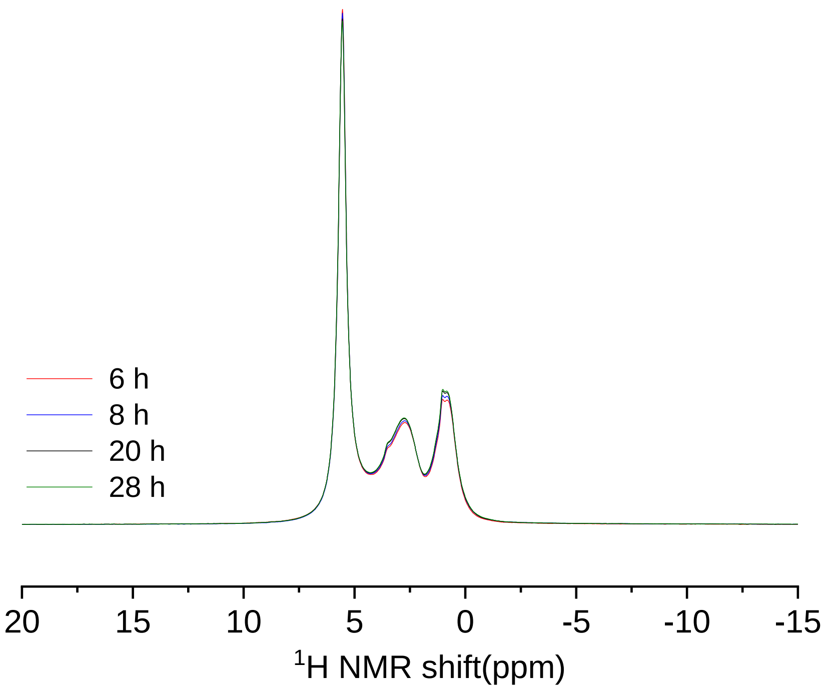

Supplementary Figure 10. The comparison of in-situ ^{1}H NMR spectra of rehydrated LDO obtained at *t* = 6, 8, 20 and 28 h (mass ratio of LDO to D_2O is approx. 1:1).

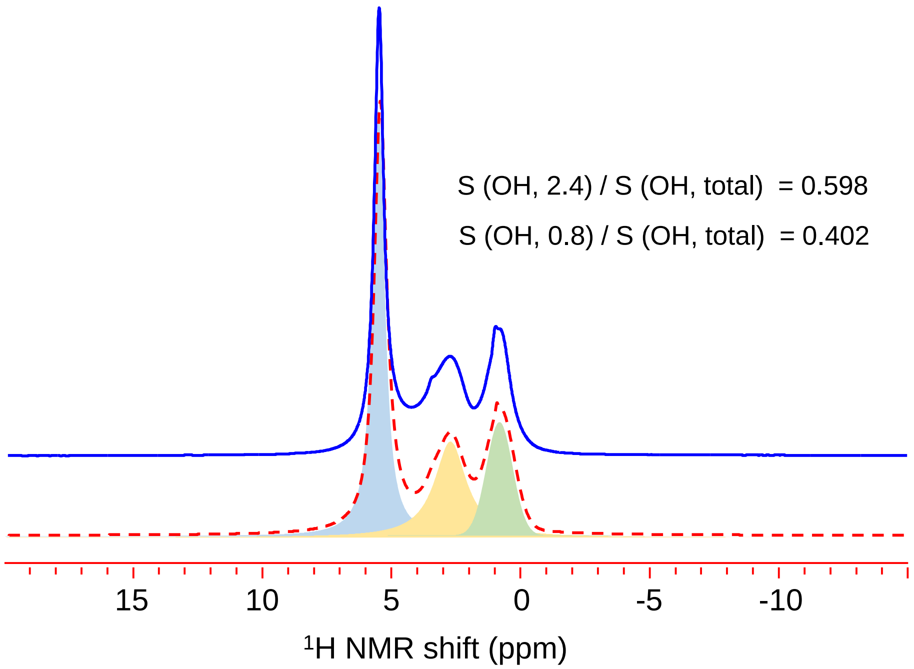

Supplementary Figure 11. In-situ ^{1}H NMR spectra of rehydrated LDO at t = 6 h with simulations decomposed into each individual component (mass ratio of LDO to D_2O is approx. 1:1).

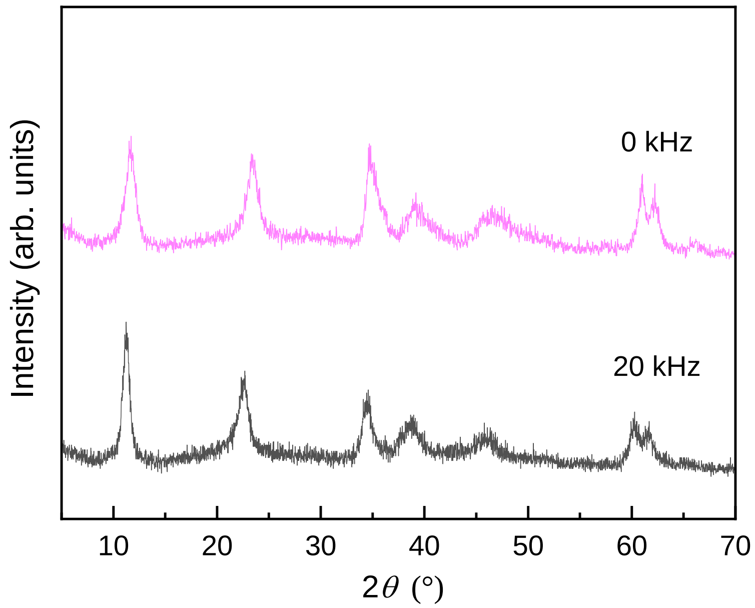

Supplementary Figure 12. XRD pattern of the sample right after a solid-state recovery process (mass ratio of Mg/Al-LDO to D_2O is approx. 1:1, rehydration time t = 6 h, MAS rate = 0 Hz or 20 kHz). This result shows that MAS rate has little effect on solid-state recovery rate.

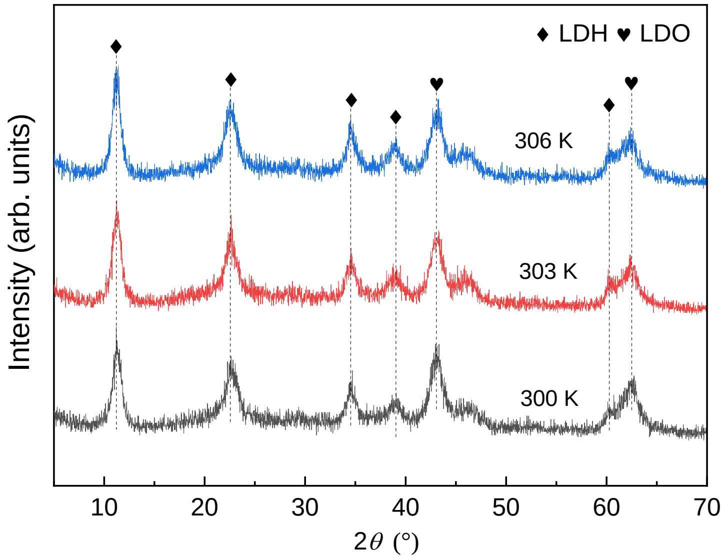

Supplementary Figure 13. XRD patterns of the samples in a "conventional recovery" with rehydration time *t* of 6 h at 300, 303 and 306 K. In situ NMR data for solid-state recovery samples were collected at 303 K, while conventional recovery was performed at 300 K, leading to a 3 K difference in temperature for the two recovery processes. The peak due to LDO are still very strong in the XRD pattern of the sample in conventional recovery with rehydration time of 6 h at 306 K. Therefore the 3 K difference in temperature is not likely to be the major reason that solid-state recovery is several times faster than conventional recovery.

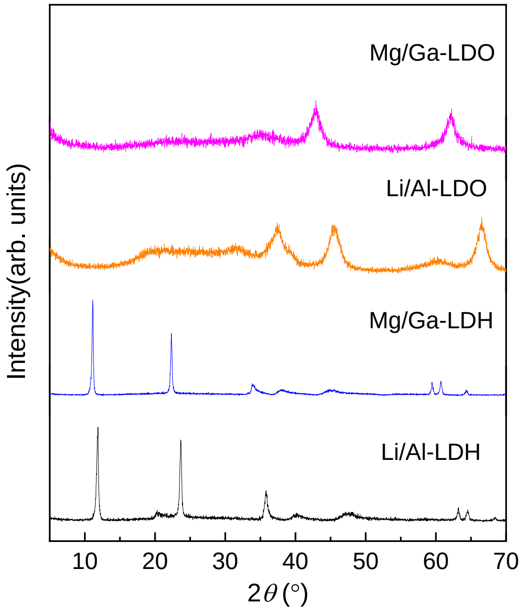

Supplementary Figure 14. XRD patterns of parent Li/Al-LDH and Mg/Ga-LDH, as well as the corresponding thermal treatment product Li/Al-LDO and Mg/Ga-LDO. These results are in agreement with the literature.[6, 7]

Supplementary Tables

Supplementary Table 1. The composition of the parent Mg/Al-LDH sample according to elemental analyses and TG analyses.

Sample	Chemical formula
Mg/Al-LDH	$Mg_{0.802}Al_{0.198}(OH)_{2.000}(NO_3)_{0.198}\cdot 0.5H_2O$

Supplementary Table 2. Lattice parameters extracted from cell refinements according to the XRD data (Fig. 2), in comparison to the parent Mg/Al-LDH.

t	2θ (003) / °	2θ (110) / °	a / Å	c / Å
10 min	-	-	-	-
30 min	11.81	-	-	22.44
1 h	11.72	-		22.63
2 h	11.65	-		22.75
6 h	11.58	-		22.89
12 h	11.47	-		23.12
20 h	11.26	60.18	3.06	23.55
28 h	11.16	60.12	3.07	23.77
36 h	11.08	60.03	3.08	23.91
48 h	11.08	60.03	3.08	23.91
Mg/Al-LDH	11.08	60.03	3.08	23.91

“-”: Not detected.

Supplementary Table 3. The mass percentage of nitrate in original Mg/Al-LDH and rehydrated Mg/Al-LDO in a conventional recovery with rehydration time *t* of 6, 12, 20, 28 and 36 h.

	6 h	12 h	20 h	28 h	36 h	Mg/Al-LDH
N (%)	0.42	0.89	1.97	3.12	3.42	3.43
NO_3^- (%)	1.86	3.94	8.72	13.37	15.15	15.19

Supplementary Table 4. The cation concentration in the supernatant after centrifugation of the sample in a conventional recovery with rehydration time t of 0 min, 60 min, 360 min (6 h), 1200 min (20 h), 1680 min (28 h) and 2160 min (36 h).

Cation	0 h	1 h	6 h	20 h	28 h	36 h
Mg (mg/L)	0	4.33	3.48	3.16	2.78	0
Al (mg/L)	0	0.79	0.58	0.32	0	0
Mg/Al (mol/mol)	-	6.2	6.9	10.9	-	-

"-": Not detected.

Supplementary References

1. Kresse G, Furthmu¨ller J. Efficient iterative schemes for ab initio total-energy calculations using a plane-wave basis set. *Phys. Rev. B* **54**, 169-186 (1996).
2. Perdew JP, Burke K, Ernzerhof M. Generalized Gradient Approximation Made Simple. *Phys. Rev. Lett.* **77**, 3865-3868 (1996).
3. Sideris PJ, Nielsen UG, Gan Z, Grey CP. Mg/Al Ordering in Layered Double Hydroxides Revealed by Multinuclear NMR Spectroscopy. *Science* **321**, 113-117 (2008).
4. Yu G*, et al.* Dehydration and Dehydroxylation of Layered Double Hydroxides: New Insights from Solid-State NMR and FT-IR Studies of Deuterated Samples. *J. Phys. Chem. C* **119**, 12325-12334 (2015).
5. Kameda T, Fubasami Y, Uchiyama N, Yoshioka T. Elimination behavior of nitrogen oxides from a NO_3^--intercalated Mg–Al layered double hydroxide during thermal decomposition. *Thermochim. Acta* **499**, 106-110 (2010).
6. Petersen LB, Lipton AS, Zorin V, Nielsen UG. Local environment and composition of magnesium gallium layered double hydroxides determined from solid-state ^{1}H and ^{71}Ga NMR spectroscopy. *J. Solid State Chem.* **219**, 242-246 (2014).
7. Graham TR*, et al.* Unraveling Gibbsite Transformation Pathways into LiAl-LDH in Concentrated Lithium Hydroxide. *Inorg. Chem.* **58**, 12385-12394 (2019).
